# Parental Resilience and Adolescent Mental Well-Being: A Population-Based Study

**DOI:** 10.3390/children13050615

**Published:** 2026-04-29

**Authors:** Christian J. Wiedermann, Verena Barbieri, Giuliano Piccoliori, Doris Hager von Strobele Prainsack

**Affiliations:** Institute of General Practice and Public Health, Claudiana College of Health Professions, 39100 Bolzano, Italy

**Keywords:** adolescence, parental resilience, perceived social support, family resources, mental well-being, population-based study, brief resilience scale, South Tyrol

## Abstract

**Highlights:**

**What are the main findings?**

- Parental resilience was independently associated with better health-related quality of life and fewer emotional, depressive, and anxiety symptoms in adolescents aged 11–19, after adjusting for perceived social support and sociodemographic factors.
- Adolescent-perceived family support did not mediate this association, and no moderation by gender or developmental stage was observed, suggesting that parental resilience and adolescent social support operate as parallel psychosocial resources.

**What are the implications of the main findings?**

- Parental psychological resources warrant consideration alongside adolescent-level factors in population-based monitoring of adolescent mental health.
- Interventions aimed at strengthening parental resilience may complement existing approaches that target adolescent social relationships and coping, though longitudinal research is needed to establish directionality.

**Abstract:**

**Background/Objectives:** Adolescence is a critical period in terms of mental health, with the family environment being a key determinant. Parental resilience, the ability to adapt and recover from stress, is a parental psychological resource that may shape the family context of adolescent development but population-based evidence is scarce. This study examined if parental resilience is linked to adolescent mental well-being, mediated by perceived family support, and whether it varies by sex or developmental stage. **Methods:** This population-based cross-sectional study analyzed data from 2004 adolescents aged 11–19 years from the COP-S Wave 4 survey in Italy. Parental resilience was assessed using a Brief Resilience Scale. Perceived social support was measured using the Multidimensional Scale of Perceived Social Support (MSPSS), and mental well-being was assessed across five outcomes: health-related quality of life (KIDSCREEN-10), emotional difficulties (SDQ), depressive symptoms (PHQ-2), anxiety symptoms (SCARED), and psychosomatic complaints (HBSC-SCL). Regression models were used to examine associations, and mediation analyses were conducted using the PROCESS macro with bootstrap confidence intervals (5000 resamples). **Results:** Parental resilience was independently associated with better health-related quality of life, lower emotional and behavioral difficulties, fewer depressive and anxiety symptoms, and fewer psychosomatic complaints, after adjusting for adolescent social support and demographics. Parental resilience showed weak positive associations with the MSPSS subscales; the hypothesis of the strongest family support association was unsupported. The analyses did not support family support as a mediator and no moderation by sex or development was found. **Conclusions:** In this population-based sample, parental resilience was associated with multiple dimensions of adolescent mental well-being that were distinct from adolescents’ perceptions of social support. These findings suggest that strengthening parental resilience may promote adolescent mental health at the population level.

## 1. Introduction

Adolescent mental health is a major public health concern in Europe. The prevalence of mental disorders and psychological distress has increased over the past decades. European community studies found that 15% of children and adolescents meet criteria for mental disorders, primarily anxiety and depression [1]. Studies show rising psychological distress and declining well-being, particularly among adolescent girls [2,3,4,5]. WHO Europe reports increased internalizing symptoms, self-harm, and neurodevelopmental diagnoses among youth [6]. Many adolescents with significant symptoms lack mental health care [7]. Untreated problems predict impairments in education, social functioning, employment, and health [1,8]. This indicates that adolescent mental health is a broader public health challenge beyond specialized services.

As adolescents focus on peers beyond family, social support from multiple sources affects mental well-being. High-quality friendships with trust and low conflict link to greater life satisfaction, happiness, and well-being, while reducing depression and loneliness [9]. Longitudinal research shows that peer support quality, not friend quantity, predicts fewer depressive symptoms into adulthood [10,11]. Studies examining multiple support sources demonstrate that peers, family, and other relationships independently contribute to adolescent mental health, varying by developmental stage [12,13]. For vulnerable adolescents, higher perceived support from friends, family, or trusted adults correlates with better mental health outcomes [12,14]. Thus, adolescent mental well-being depends on support across multiple relationships rather than a single source.

Parental resilience, the capacity to adapt and recover from stress, is a parental psychological resource that may shape the family context of child development [15]. Family resilience frameworks show that parental coping affects family functioning and parent–child relationships [16,17]. Resilient parents maintain responsive caregiving under stress, creating supportive environments for children [18]. These protective resources operate through the overall family climate that parents establish [16].

Empirical evidence shows that parental psychological resources, including resilience and mental health, relate to adolescents’ perceived social support within families [19]. Adolescents who perceive their parents as emotionally available report higher family support, leading to better mental health [20]. Parental resilience may indirectly promote adolescent well-being by enabling reliable family support [18,19]. However, few population-based studies examine parental resilience as a predictor of adolescent social support and mental health [21].

Despite recognizing the potential relevance of parental resilience for adolescent mental health, evidence linking it to adolescent outcomes in the general population is limited. Studies on parental influences use cross-sectional designs, limiting causal inference but showing how family resources relate to adolescent mental health [11,22,23]. In longitudinal research, parental mental health is often a covariate, not a primary focus [4,5]. Parental resilience is inconsistently defined, and tools like the Brief Resilience Scale are rarely used in family surveys [22,24,25]. Most evidence comes from clinical or high-risk samples, with few population-based studies in Europe [22,26]. Population-based evidence examining parental resilience alongside adolescent social support and mental well-being outcomes remains limited, particularly in European settings.

This study examined relationships between parental resilience, adolescent social support, and adolescent mental well-being during the ages of 11–19 years.

- We examined whether parental resilience was associated with adolescent mental well-being outcomes, namely health-related quality of life (HRQoL), emotional and behavioral difficulties (SDQ total difficulties), depressive symptoms (PHQ-2), anxiety symptoms (SCARED), and psychosomatic complaints (HBSC-SCL), after adjusting for sociodemographic factors and adolescent social support. These outcomes represented positive functioning and common symptom domains in adolescent mental health.
- Second, we tested whether parental resilience was differentially associated with adolescents’ perceived support from family, friends, and significant others. Based on family resilience models [16,17], we hypothesized that parental resilience would show stronger association with family support than peer support, reflecting its influence on the home environment.
- Third, we examined whether adolescent-perceived family support mediated the link between parental resilience and adolescent mental well-being. Research suggests parental psychological resources may indirectly promote adolescent well-being through supportive family relationships [18,22]. We tested this mediation model using bias-corrected bootstrap confidence intervals [23].
- Fourth, we explored whether parental resilience and adolescent mental well-being associations varied by gender and developmental stage (early vs. late adolescence). Research shows that family relationships’ importance differs by gender [4,24] and developmental stage [10,13,25], remaining especially relevant for younger adolescents with distinct patterns between boys and girls.

Although the cross-sectional design limits causal inference, this study extends the existing literature by examining parent-reported resilience alongside adolescent-perceived social support and multiple mental well-being outcomes in a European population-based sample.

## 2. Materials and Methods

### 2.1. Setting, Study Design and Sample

This study used data from the fourth wave of the Corona and Psyche—South Tyrol (COP-S) survey, a population-based monitoring project examining psychosocial well-being among children and adolescents in South Tyrol, Italy [26,27]. The fourth wave was conducted as an anonymous online survey between 17 March and 13 April 2025, using a repeated cross-sectional design.

Families with school-aged children in South Tyrol were invited to participate through the provincial public school system. Families with more than one adolescent were asked to respond for only one child; however, compliance could not be verified. The anonymous design did not record school affiliation or allow for identification of multiple responses from the same household; family-level and school-level clustering could therefore not be assessed. Public school directorates distributed individualized survey links via email to all families who had provided email addresses to the school system. Approximately 40,000 families were contacted; however, precise enrollment numbers were not available. A total of 9734 families participated, with 7818 (80.3%) providing evaluable responses, corresponding to an estimated participation rate of approximately 24% (7818/~40,000). Among the evaluable responses, 2554 adolescents aged 11–19 years (32.7% of evaluable parent questionnaires) completed the parallel self-report questionnaire.

Data were collected using the SoSci Survey platform (version 3.2.46; SoSci Survey GmbH, Munich, Germany). The fourth wave maintained the core structure and validated instruments from earlier COP-S waves [26,27] but expanded to include psychosocial resources, perceived social support, parental resilience, health literacy, and post-pandemic stressors relevant to adolescent mental well-being.

Adolescents self-reported all mental well-being outcomes (HRQoL, SDQ total difficulties, depressive symptoms, anxiety symptoms, and psychosomatic complaints) and perceived social support. One parent or legal guardian per adolescent completed a parallel questionnaire providing sociodemographic information, family structure, socioeconomic status, migration background, language, and parental resilience. We used parental reports for sociodemographic background variables and to assess parental resilience as the primary independent variable.

Participation required informed consent from a parent or legal guardian and assent from the adolescent. The analytic sample closely reflected the regional distribution of age and sex among South Tyrol adolescents, according to official provincial statistics. Given the multilingual context of South Tyrol, we collected information on family language (reported by parents) and used it descriptively but not as a primary exposure variable or analytical covariate. Health literacy was likewise treated as a sample descriptor rather than an analytical covariate, as its associations with both socioeconomic position and mental well-being outcomes could introduce collider bias.

### 2.2. Measures

#### 2.2.1. Sociodemographic and Contextual Variables

Participants self-reported their age (in years) and sex. Parents provided information on the school language (German, Italian, Ladin), family language spoken at home, and school level.

We classified the place of residence as urban or rural according to official provincial administrative criteria. Parents reported their education levels, which we categorized using the Comparative Analysis of Social Mobility in Industrial Nations (CASMIN) framework as low (primary or lower secondary), medium (upper secondary), or high (tertiary education) [28].

We classified household structure as two-parent or single-parent households. We coded migration background as a binary variable indicating whether at least one parent was born outside Italy. We assessed family socioeconomic status using the Family Affluence Scale III (FAS III), a validated six-item measure of material assets and family living conditions [29,30]. Total scores range from 0 to 13, with higher values indicating higher affluence. We used FAS III primarily as a continuous covariate and categorized it into low, medium, and high affluence for descriptive purposes [31].

We evaluated adolescent health literacy using the 10-item Health Literacy for School-Aged Children (HLSAC) tool [32]. This instrument measures perceived difficulty in obtaining, understanding, evaluating, and using health-related information on a 4-point Likert scale from “very difficult” (1) to “very easy” (4). The total scores range from 10 to 40, with higher scores indicating greater health literacy. The HLSAC has strong psychometric properties for adolescents. The German version shows unidimensionality, high internal consistency (Cronbach’s α = 0.88), and measurement invariance based on HBSC data [33]. The Italian version (HLSAC-I) demonstrated good reliability and construct validity in representative adolescent samples [34].

#### 2.2.2. Perceived Social Support

We measured perceived social support using the Multidimensional Scale of Perceived Social Support (MSPSS), a validated 12-item instrument that assesses perceived support from family, friends, and significant others [35]. Items are rated on a 7-point Likert scale from 1 (“very strongly disagree”) to 7 (“very strongly agree”), with higher scores indicating greater perceived social support. We calculated mean scores for the total scale and each subscale.

The MSPSS demonstrates good reliability and construct validity across adolescent populations and language versions, including the German and Italian samples [36,37]. We used the total score as the primary indicator of perceived social support and examined the subscale scores in secondary analyses.

#### 2.2.3. Resilience

We assessed parental resilience using the Brief Resilience Scale (BRS), a six-item self-report instrument that conceptualizes resilience as the capacity to recover or “bounce back” from stress, rather than as a stable personality trait [15]. In the COP-S Wave 4 survey, the BRS was administered to the parent or legal guardian who completed the parallel questionnaire alongside the adolescent self-report. Parental resilience was therefore measured as a parent-reported construct reflecting the responding parent’s own perceived recovery capacity. Items are rated on a five-point Likert scale from 1 (“strongly disagree”) to 5 (“strongly agree”). Negatively worded items (items 2, 4, and 6) were reverse-coded prior to scale construction. We calculated a mean score across all six items, with higher values indicating greater parental resilience. Cases with missing responses on one or more items were excluded from scale computation (n = 36).

The BRS has good reliability and construct validity in clinical and population-based adult samples [15]. In a German population-based validation study, good internal consistency (Cronbach’s α ≈ 0.85), a largely unidimensional structure accounting for wording effects, and meaningful associations with mental health, social support, and coping indicators [38]. An Italian validation study in adult and community samples reported satisfactory psychometric properties, including good internal consistency and construct validity [39]. These validation studies support the use of the BRS in German- and Italian-speaking adult populations, as represented by the parent sample in South Tyrol.

In the present study, parental resilience served as the primary independent variable. We examined its associations with adolescent-reported mental well-being outcomes and adolescents’ perceived social support, treating parental resilience as a parental psychological resource operating within the family context rather than an adolescent characteristic.

#### 2.2.4. Mental Well-Being Outcomes of Adolescents

We measured HRQoL using the 10-item KIDSCREEN questionnaire (KIDSCREEN-10), a generic instrument covering the physical, psychological, and social aspects of well-being in children and adolescents. Items were transformed into standardized T-scores (mean 50, SD 10) using Rasch modeling, with higher scores indicating better perceived quality of life [40].

We assessed emotional and behavioral difficulties using the total difficulty score of the Strengths and Difficulties Questionnaire (SDQ). The SDQ total score reflects overall psychosocial difficulties, with higher scores indicating a greater symptom burden.

We assessed depressive symptoms using the Patient Health Questionnaire-2 (PHQ-2), a two-item screening instrument rated on a 4-point scale from 0 (“not at all”) to 3 (“nearly every day”). Higher scores indicate more severe depressive symptoms [41,42,43].

Anxiety symptoms were measured using the Generalized Anxiety Disorder subscale of the Screen for Child Anxiety-Related Emotional Disorders (SCARED-GAD). The nine items are rated on a 3-point scale from 0 (“not true”) to 2 (“very or often true”), with higher scores reflecting greater anxiety symptoms [44,45,46].

We assessed psychosomatic complaints using the Health Behavior in School-aged Children Symptom Checklist (HBSC-SCL), an eight-item instrument capturing the frequency of common somatic and emotional complaints in the past week (headaches, stomachaches, backaches, feeling low, irritability, nervousness, sleep difficulties, and dizziness). Items are rated on a 5-point frequency scale ranging from 1 (daily) to 5 (not at all) [47]. We computed the number of different complaints reported at least once per week (range 0–8), with higher scores indicating a greater complaint burden.

#### 2.2.5. Internal Consistency

We assessed the internal consistency of all multi-item scales using Cronbach’s alpha (α) based on complete cases for each instrument. Perceived social support, measured using the 12-item MSPSS, showed excellent internal consistency (α = 0.98). The MSPSS subscales also showed high internal consistency: family support (α = 0.97), friend support (α = 0.97), and significant-other support (α = 0.97). Adolescent health literacy, assessed using the 10-item HLSAC, showed good internal consistency (α = 0.90). Parental resilience, assessed using the 6-item BRS administered to parents, showed good internal consistency (α = 0.85).

### 2.3. Statistical Analysis

A sample size of approximately 1500 adolescents with complete data across the primary analysis variables offers sufficient statistical power to identify small (Cohen’s d ≈ 0.20) to moderate (Cohen’s d ≈ 0.50) group differences at α = 0.05 in analyses of means and proportions, as outlined in standard power analysis frameworks [48]. No post-stratification or statistical weighting was conducted. All analyses were conducted using IBM SPSS Statistics (version 27.0; IBM Corp., Armonk, NY, USA), with statistical significance set at *p* < 0.05 (two-tailed).

#### 2.3.1. Descriptive and Correlation Analyses

Descriptive statistics were used to summarize sample characteristics, psychosocial resources, and mental well-being indicators. Categorical variables are reported as absolute frequencies and percentages, and continuous variables are reported as means with standard deviations (SDs). The observed score ranges and valid sample sizes are reported for the outcome measures.

Zero-order Spearman rank-order correlation coefficients were calculated to examine the bivariate associations between perceived social support (MSPSS total score and subscales) and parental resilience (BRS mean score) and adolescent mental well-being outcomes. Correlation analyses were based on the available case pairs for each variable combination.

#### 2.3.2. Regression Analyses

Associations between parental resilience, perceived social support, and adolescent mental well-being were examined using hierarchical multivariate linear regression models. Models were fitted for five outcomes: HRQoL, emotional and behavioral difficulties (SDQ total difficulties score), depressive symptoms (PHQ-2), anxiety symptoms (SCARED), and psychosomatic complaints.

Three hierarchical models were estimated for each outcome. Model 1 included sociodemographic covariates only, establishing a baseline-adjusted association. Model 2 added perceived social support (MSPSS total score) to assess its contribution beyond sociodemographic factors. Model 3 further added parental resilience (BRS mean score) to assess its independent association with adolescent mental well-being and its incremental contribution beyond both covariates and perceived social support.

The sociodemographic covariates in all models were adolescent age (continuous), sex, parental education (CASMIN-compatible categories), family affluence (FAS III, continuous), migration background, and household structure (single- vs. two-parent household). Perceived social support was operationalized as the total score on the MSPSS. Parental resilience was included as a continuous variable (BRS mean score, as described in Section 2.2.3).

Regression coefficients are reported as unstandardized estimates with 95% confidence intervals and *p*-values. Standardized coefficients (β) are also reported to facilitate comparisons of effect sizes across outcomes. Changes in explained variance (ΔR^2^) between successive models assessed the incremental contribution of each predictor block.

Variance inflation factors (VIFs) were examined for multicollinearity; all values were below the conventional threshold (maximum VIF = 1.03 across all models), indicating no collinearity concerns.

Missing data were handled using listwise deletion within each regression model; no imputation procedures were performed. Analytic sample sizes varied between approximately 1462 and 1527 depending on the outcome and are reported for each model in the respective tables. Descriptive statistics in Table 1 are based on all available cases per variable, with valid n reported for each variable block; these denominators differ from the regression-specific analytic samples.

#### 2.3.3. Mediation and Moderation Analyses

Mediation and moderation analyses were conducted using PROCESS macro for SPSS (version 4.3). Age was retained as a continuous covariate in the developmental stage models to adjust for linear age trends within each stage group, while the dichotomous moderator tested differences in the resilience–outcome association between early and late adolescence. Models were estimated using bias-corrected bootstrap confidence intervals with 5000 resamples. Coefficients are reported as unstandardized regression estimates. Given the cross-sectional design, mediation results were interpreted as statistical associations rather than causal pathways.

Simple mediation models examined whether adolescents’ perceived family support mediated the association between parents’ resilience and mental well-being outcomes. Parents’ resilience served as the independent variable, family support as mediator, and mental well-being as dependent variable. Models were adjusted for age, sex, parental education, family affluence, migration background, household composition, and urbanicity. Mediation was confirmed when the 95% bootstrap confidence interval for indirect effect excluded zero.

Moderation analyses using PROCESS Model 1 tested whether parental resilience–adolescent mental well-being associations varied by sex and developmental stage. Models were estimated with sex (female vs. male) and developmental stage (early adolescence: 11–14 years, coded 0; late adolescence: 15–19 years, coded 1) as moderators. Models included covariates of age, parental education, family affluence, migration background, household composition, and urbanicity, with sex added for developmental stage models. Significant interactions (*p* < 0.05) were analyzed using simple slopes; otherwise, main effects are reported.

### 2.4. Use of Generative Artificial Intelligence Tools

Generative artificial intelligence (AI) tools, particularly ChatGPT-4 from OpenAI (San Francisco, USA), were employed to aid in organizing and refining the language in the Introduction, Methods, and Discussion sections. These AI tools were not used for data analysis or statistical calculations. Additionally, AI was used to synthesize and cross-reference the existing literature to enhance clarity and context. All content was reviewed and approved by the authors.

## 3. Results

### 3.1. Sample Characteristics

Of the 2005 adolescents who completed the self-report questionnaire, 2004 were in the predefined age range of 11–19 years and were included in the analytic sample. The descriptive characteristics of the analytic sample are presented in Table 1, which summarizes the sociodemographic characteristics, language context, socioeconomic position, psychosocial resources, and perceived social support. The mean age was in mid-adolescence, with a slight predominance of early adolescents over late adolescents. The sex distribution was balanced. Most adolescents reported German as their family and school language, and the majority resided in rural areas.

Parental education was broadly distributed across the CASMIN categories, with comparable proportions in the medium- and high-education groups and a smaller share in the low-education category. Most adolescents reported no migration background and lived in two-parent households. Family affluence was predominantly in the medium range, whereas low- and high-affluence groups accounted for smaller proportions of the sample.

The descriptive levels and distributions of adolescent health literacy, resilience, and perceived social support are shown in Table 1. The average health literacy score was in the moderate-to-high range. Parental resilience, assessed via the BRS, showed a mean score of 3.43 (SD 0.76), spanning the full response range (1–5) with adequate dispersion across the sample. Perceived social support was generally high, with most adolescents reporting high overall support on the MSPSS of Perceived Social Support. The MSPSS total score covered nearly the full-scale range (observed range: 1–7) and had a mean that was well above the theoretical midpoint. The mean levels of perceived support were similarly elevated across the family, friend, and significant other subscales. However, the distribution of friends’ support was wider than that of family or significant others’ support, with a higher proportion of adolescents reporting moderate or low support in the peer domain, indicating greater variability in peer-related support.

### 3.2. Distribution of Well-Being Indicators and Zero-Order Correlations

The descriptive statistics for the mental well-being indicators are presented in Table 2. HRQoL showed a broad distribution, with scores spanning most of the possible range and a mean value close to the international reference value. Emotional and behavioral difficulties, depressive symptoms, and anxiety symptoms likewise demonstrated substantial variability, with most adolescents reporting low-to-moderate symptom levels and smaller proportions reporting higher symptom burdens. Psychosomatic complaints also covered a wide range, indicating meaningful heterogeneity in psychosomatic complaint-related well-being. Across all indicators, missingness was moderate and variable-specific; however, the sample sizes remained sufficient to support subsequent multivariate analyses. All well-being indicators displayed substantial interindividual variability, supporting their use as continuous variables.

The zero-order correlations between perceived social support, resilience, and well-being outcomes are presented in Supplementary Table S1. Perceived social support showed small-to-moderate associations with well-being indicators. Higher overall support was associated with better HRQoL (rs ≈ 0.44) and fewer emotional and behavioral difficulties (rs ≈ −0.37), depressive symptoms (rs ≈ −0.31), and anxiety symptoms (rs ≈ −0.25). Associations were consistently strongest for friend support, followed by family support, whereas support from significant others showed weaker associations across outcomes.

In contrast, parental resilience was only weakly correlated with perceived social support and well-being indicators (|rs| ≤ 0.17). The well-being measures were strongly interrelated, with large correlations between emotional and behavioral difficulties, depressive symptoms, anxiety symptoms, and HRQoL (|rs| ≥ 0.54). Psychosomatic complaints were negatively associated with all measures of social support and parental resilience, and positively correlated with depressive and anxiety symptoms (|rs| = 0.58–0.59).

Overall, the correlation pattern indicated that perceived social support was consistently related to adolescent well-being, whereas parental resilience was only weakly correlated at the zero-order level.

### 3.3. Parental Resilience, Perceived Social Support, and Adolescent Mental Well-Being

#### 3.3.1. Associations Between Parental Resilience and Perceived Social Support

The results of the MVLR models examining the associations between parental resilience and adolescents’ perceived social support are presented in Table 3. After adjusting for adolescent age, sex, migration background, parental education, household structure, and family affluence, higher parental resilience was independently associated with higher perceived social support across all three MSPSS subscales. The association was not restricted to the family support subscale but extended to friend support and support from significant others, with the strongest effect observed for significant-other support (β = 0.086, *p* < 0.001), followed by friend support (β = 0.077, *p* = 0.002), and family support (β = 0.051, *p* = 0.042). All associations were statistically significant but of small magnitude (ΔR^2^ ≤ 0.007 across subscales).

#### 3.3.2. Parental Resilience and Adolescent Mental Well-Being Results

A consolidated overview of the hierarchical regression results across all five mental well-being outcomes is presented in Table 4, with detailed coefficients reported in Supplementary Tables S2–S6.

In Model 1, which included sociodemographic covariates only, age, sex, and selected socioeconomic indicators showed heterogeneous associations with mental well-being outcomes. These associations were generally small and not uniform across outcomes. In Model 2, the addition of perceived social support (MSPSS total score) was associated with consistent improvement in model fit across all outcomes, with higher perceived social support being independently associated with better HRQoL and lower levels of emotional and behavioral difficulties, depressive symptoms, and anxiety symptoms. In Model 3, the further addition of parental resilience resulted in consistent improvement in model performance across all five outcomes. Higher parental resilience was independently associated with better HRQoL (B = 2.05, 95% CI [1.43; 2.67], β = 0.155, *p* < 0.001), fewer emotional and behavioral difficulties (B = −1.11, 95% CI [−1.48; −0.74], β = −0.149, *p* < 0.001), fewer depressive symptoms (B = −0.20, 95% CI [−0.28; −0.12], β = −0.115, *p* < 0.001), and fewer anxiety symptoms (B = −0.84, 95% CI [−1.14; −0.53], β = −0.135, *p* < 0.001). Higher parental resilience was also associated with fewer psychosomatic complaints (B = −0.54, 95% CI [−0.69; −0.39], β = −0.176, *p* < 0.001). The inclusion of parental resilience resulted in modest but consistent attenuation of social support coefficients, while perceived social support remained statistically significant across all five outcomes. This pattern indicates partial overlap between the two psychosocial resources while supporting their independent associations within the same models.

### 3.4. Mediation and Moderation Results

#### 3.4.1. Mediation Analyses

Formal mediation analyses did not support perceived family support as a mediator of the association between parental resilience and any adolescent mental well-being outcome. The a-path coefficient (parental resilience → MSPSS family subscale) was not significant across all models (*p* = 0.215–0.322). Although Table 3 shows a marginally significant bivariate association between parental resilience and the MSPSS family subscale (B = 0.10, *p* = 0.042), the PROCESS mediation models use listwise deletion across all model variables including each respective outcome, resulting in smaller analytic samples (*n* = 1493–1527) than the regression in Table 3 (*n* = 1639–1649). This modest reduction in sample size was sufficient to attenuate the already marginal association below the significance threshold. Accordingly, all estimated indirect effects were negligible, and bias-corrected bootstrap confidence intervals (5000 resamples) included zero in every case. The direct effects of parental resilience on mental well-being outcomes remained significant for four of the five outcomes (Supplementary Table S7). These findings indicate that parental resilience and adolescent-perceived family support operate as independent rather than sequentially linked psychosocial resources.

#### 3.4.2. Moderation Analyses

Moderation analyses examined whether adolescent sex and developmental stage modified the association between parental resilience and mental well-being. Across all five outcomes, no statistically significant interaction effects were observed, for sex (all *p* ≥ 0.176, ΔR^2^ ≤ 0.001) or for developmental stage (all *p* ≥ 0.345, ΔR^2^ ≤ 0.001; Supplementary Table S8). Therefore, the association between parental resilience and adolescent mental well-being was consistent across boys and girls and across early (ages 11–14) and late adolescence (ages 15–19).

## 4. Discussion

The findings of this study support the interpretation that parental resilience and adolescent perceived social support are parallel, independently operating psychosocial resources, with the association between parental resilience and adolescent mental health being consistent across demographic subgroups. In this population-based study of adolescents aged 11–19 years, parental resilience was associated with better HRQoL, emotional and behavioral difficulties, depressive symptoms, and anxiety symptoms, after adjusting for adolescent perceived social support and demographics. Parental resilience showed positive associations with adolescents’ perceived support from MSPSS sources—family, friends, and significant others—though effect sizes were small, with family support showing the weakest association. Mediation analyses found that adolescent-perceived family support did not statistically mediate the relationship between parental resilience and mental well-being outcomes. No evidence suggested that associations between parental resilience and adolescent mental well-being varied by sex or developmental stage. These findings indicate parental resilience is associated with adolescent mental well-being through pathways not fully captured by perceived family support in this cross-sectional study.

### 4.1. Perceived Social Support and Adolescent Mental Well-Being

Adolescent-perceived social support was associated with better mental well-being across health-related quality of life, emotional and behavioral difficulties, depressive and anxiety symptoms, and psychosomatic complaints, after adjusting for sociodemographic characteristics. The effect sizes were small to moderate [24,49]. These associations remained after including parental resilience, indicating social support independently correlates with mental well-being beyond parental psychological resources.

Among MSPSS subscales, friend support showed the strongest association with mental well-being, followed by family support, with significant others showing weaker associations. This aligns with evidence that peer relationships become increasingly important during adolescence [13,25]. The independent association of all three support sources with adolescent mental well-being indicates that perceived social support is multi-sourced, and analyzing single support domains may underestimate its relationship with well-being [12,13].

The consistency of associations across diverse well-being indicators suggests that perceived social support relates broadly to adolescents’ psychological functioning rather than to isolated symptom domains. International population-based surveys have documented associations between perceived social support and multiple outcomes, including life satisfaction, emotional distress, psychosomatic complaints, and suicidality, supporting its relevance across the full spectrum of adolescent mental health [24,49]. The modest effect sizes are consistent with population studies showing that perceived social support accounts for limited variance in mental health outcomes when sociodemographic and other psychosocial factors are simultaneously considered [50].

### 4.2. Parental Resilience and Adolescent Mental Well-Being

Parental resilience was associated with all five adolescent mental well-being outcomes, HRQoL, emotional and behavioral difficulties, depressive symptoms, anxiety symptoms, and psychosomatic complaints, after adjusting for perceived social support and sociodemographic factors. The small but consistent effect sizes across all outcomes indicate that parental resilience is a meaningful family-level correlate of adolescent mental well-being.

These findings align with theoretical models proposing that parental psychological resources shape the family context in which child development occurs [16,17]. Parents who better adapt to stress can maintain emotional availability and consistent caregiving, supporting adolescent psychological well-being [18]. This suggests parental resilience correlates with adolescent mental health across the general population, not just in high-risk families.

Prior research has examined parental psychological resources in clinical populations or families facing adversities [16,51]. These findings indicate parental resilience’s importance in the broader population, supporting its inclusion in population-based family health monitoring. European population-based studies on parental psychological resources and adolescent mental health are limited [51,52], and these results help address this gap.

### 4.3. Parental Resilience and Adolescent Social Support

This study examines parental and adolescent psychosocial resources in relation to adolescent mental well-being by modeling parental resilience alongside adolescent perceived social support. The results showed both factors maintained independent associations with adolescent mental well-being, with parental resilience explaining additional variance beyond sociodemographic factors and social support. This indicates parental resilience represents a distinct parental resource dimension not captured by adolescents’ social relationship experiences.

Parental resilience showed positive associations with adolescents’ perceived support across all MSPSS sources, not just family support. This suggests a broader connection between parental psychological resources and adolescents’ ability to perceive social support from multiple sources. This aligns with models suggesting that resilient family environments foster social competence and positive relational schemas in adolescents [16,17]. The specificity hypothesis linking parental resilience primarily to family support was not supported, with modest associations across subscales and strongest correlation with significant others.

Mediation analyses did not support adolescent-perceived family support as a pathway linking parental resilience to mental well-being outcomes, as the path from parental resilience to family support was not statistically significant. This null finding aligns with prior research: cross-sectional designs capture concurrent associations rather than developmental processes that may require longitudinal assessment [23]. Additionally, adolescent self-reported family support may not fully capture the parenting behaviors through which parental resilience operates [18]. Studies have shown that family support and psychological functioning show parallel rather than sequential associations with adolescent depression [23], consistent with our findings.

The absence of moderation by gender and developmental stage showed that parental resilience and adolescent mental well-being association did not differ across subgroups. While research suggests family relationships vary by gender and stage [4,12,25], findings indicate parental resilience is broadly associated with adolescent mental well-being rather than being specific to particular demographic subgroups. Community surveys show associations between parental resources and adolescent mental health across demographics [24,53], supporting this interpretation.

### 4.4. Comparison with Previous Research and Conceptual Implications

These findings extend the adolescent mental health literature by examining parental resilience as a predictor of well-being in a European population-based sample, highlighting differences from studies examining resilience as an individual characteristic. Prior research frames resilience as an intrapersonal adolescent resource associated with mental health outcomes [52,53]. This study instead treats resilience as a parental attribute whose associations with adolescent outcomes are examined within a family context framework [16,17].

Family resilience theory differentiates between individual resilience (capacity to recover from adversity) and family resilience (processes through which families maintain functioning under stress) [16]. A resilient parent’s adaptive capacity contributes to family stability. Our findings show parental resilience is independently associated with adolescent mental well-being beyond adolescent-perceived social support, indicating a broader relationship than captured by explicit family support alone.

The directionality assumed in this study—parental resources → family environment → adolescent outcomes—has empirical support, though rarely tested with parental resilience as the primary exposure. Research shows family and peer support act as resilience-supporting resources for adolescents, with reciprocal associations with depressive symptoms [23]. Structural equation models indicate parental and social resources enhance adolescent coping, linking to mental health outcomes [22]. These cross-sectional findings align with existing evidence while adding a population-level perspective.

The lack of statistically supported mediation through adolescent-perceived family support should be seen as consistent with the cross-sectional design rather than contradicting the theoretical model. Cross-sectional designs capture only concurrent associations and cannot effectively detect sequential developmental pathways [23]. The pathway from parental to adolescent-perceived family support may develop over time and require longitudinal measurements. The MSPSS family subscale’s subjective appraisal may not fully capture the parenting behaviors through which parental resilience operates [18]. Future multi-informant and longitudinal research would better test the mediating mechanisms in the family resilience framework.

These findings can be situated within broader population-based evidence on adolescent mental health. In a German HBSC study, social support moderated the association between loneliness and mental health in children and adolescents, confirming social resources as independent correlates of well-being in a Central European sample [54]. Data from the German COPSY study similarly showed stable associations between social support, family climate, and adolescent mental health across pandemic waves [29]. At a global level, a 82-country analysis identified family connectedness as a correlate of lower anxiety and suicidal ideation prevalence among adolescents [55]. In a Chinese longitudinal study, social support and resilience were independently associated with fewer mental health problems in early adolescence [22]. While these studies consistently identify social support and family resources as correlates of adolescent mental health, most examine resilience as an individual adolescent characteristic. The present study extends this evidence by treating resilience as a parental attribute, providing a distinct perspective on how family-level psychological resources relate to adolescent well-being.

### 4.5. Implications for Public Health and Adolescent Mental Health Promotion

These findings have implications for adolescent mental health promotion beyond conventional interventions. Parental resilience as a modifiable parental resource associated with adolescent mental well-being suggests that strengthening it may be a useful upstream strategy, complementing direct adolescent interventions.

Parenting-focused interventions have demonstrated effects on parental stress, coping, and psychological well-being. Structured parenting programs based on cognitive-behavioral and family systems approaches improve both parenting behavior and parental psychological functioning [56,57]. Group-based interventions that enhance coping, social connection, and emotional availability reduce parental distress and improve family relationships [55]. These programs’ improvements in parental resources have been linked to reduced adolescent internalizing symptoms [57].

Parental resilience may be particularly relevant for universal prevention, as its associations with adolescent mental well-being were observed across the general population. Population-based family health monitoring in Europe focuses on adolescent symptoms and social indicators, but rarely includes validated assessments of parental psychological resources [51,52]. Including brief parental resilience measures in population surveys would enable tracking of this family resource and its association with adolescent outcomes [37].

Public health initiatives increasingly recognize family environment as a determinant of adolescent mental health, particularly during societal stressors like COVID-19 [24,54]. These findings indicate parental resilience should be considered alongside family socioeconomic status and social support. Including parental resilience in monitoring frameworks may help identify families with fewer psychosocial resources and complement existing indicators of adolescent well-being.

### 4.6. Strengths and Limitations

This study has key strengths. The population-based design from a province-wide school survey enabled examining parental resilience and adolescent well-being across a broad sample. Validated instruments with established psychometric properties in German and Italian populations enhanced measurement comparability in South Tyrol’s bilingual context. The assessment of multiple well-being outcomes provided a comprehensive framework for adolescent mental health. The cross-informant design, with parents reporting resilience and adolescents reporting well-being outcomes, reduced shared method variance risk.

This study has limitations. The cross-sectional design precludes causal inference, as the directionality of parental resilience predicting adolescent mental well-being cannot be established from concurrent data. Parental resilience was assessed using a brief global scale at a single time point, potentially not capturing its dynamic nature [16,17]. The anonymous design precluded assessment of family-level or school-level clustering, which may have affected standard error estimates. Complete-case analysis assumes that data are missing at random, an assumption that cannot be verified from the observed data. Moreover, the BRS was completed by only one responding parent or guardian per family, and the study did not assess family functioning, parenting practices, or dyadic family processes directly; the interpretation of parental resilience as a contextual resource operating within the family system is therefore theoretically motivated but not empirically established by the present data. The hypothesized mediator, adolescent-perceived family support, may not adequately reflect the parenting behaviors through which parental resilience operates [18]. From 40,000 invited families, 7818 provided responses (24% participation rate), with 2004 adolescents contributing data and analytical samples varying between 1500 and 1700 across outcomes. The low participation rate suggests potential selection bias, as responding families may differ in health literacy and socioeconomic resources. Recruitment limited to email-registered families may underrepresent disadvantaged households. While the population showed comparable age and sex distributions to provincial statistics, formal representativeness analyses were not possible. Unmeasured confounders, including family mental health history and parenting quality, may influence both parental resilience and adolescent outcomes.

Despite these limitations, this study provides evidence that parental resilience is associated with adolescent mental well-being across multiple domains in a European population-based sample.

## 5. Conclusions

In this population-based study of South Tyrolean adolescents aged 11–19, parental resilience was associated with better HRQoL, lower emotional and behavioral difficulties, reduced depressive and anxiety symptoms, and fewer psychosomatic complaints, after adjusting for adolescent social support and demographics. Adolescent social support was independently linked to all mental well-being outcomes, including psychosomatic complaints. These findings show that parental resilience and adolescent social support are distinct resources independently associated with adolescent mental well-being.

The findings did not support adolescent-perceived family support as mediating parental resilience and mental well-being, or moderation by sex or developmental stage. Parental resilience appears to operate as a distal family resource, with its association with adolescent outcomes not accounted for by perceived family support, suggesting that the mechanisms linking parental resources to adolescent well-being require longitudinal investigation.

Parents’ psychological resources are independently associated with adolescent mental health at the population level, extending research beyond individual psychosocial factors. Family-based interventions to strengthen parental resilience may complement existing approaches that target adolescent relationships and coping. While causation cannot be established from cross-sectional data, this study suggests that parents’ resilience warrants consideration in research and public health strategies for adolescent mental health promotion.

## Figures and Tables

**Table 1:** Sociodemographic characteristics, language context, socioeconomic position, psychosocial resources, and perceived social support in a population-based sample of adolescents aged 11–19 years (*n* = 2004).

Characteristic	Valid *n*	*n* (%) or Mean ± SD ^1^
Age (years)	2004	14.4 ± 2.34 (range 11–19)
Adolescence stage ^2^	2004	
Early		1065 (53.1)
Late		939 (46.9)
Sex	2004	
Male		1027 (51.2)
Female		977 (48.8)
Family language ^3^	1993	
German		1631 (81.8)
Italian		285 (14.3)
Ladin		53 (2.7)
Other		24 (1.2)
School language ^3^	1993	
German		1726 (86.6)
Italian		218 (10.9)
Ladin		49 (2.5)
Residence ^4^	2004	
Rural		1433 (71.5)
Urban		571 (28.5)
Parental education (CASMIN-compatible)	1982	
Low		367 (18.5)
Medium		832 (42.0)
High		783 (39.5)
Missing		22
Migration background	1960	
No		1790 (91.3)
Yes		170 (8.7)
Household structure	1992	
Single-parent household		340 (17.2)
Two-parent household		1642 (82.8)
Family Affluence Scale (FAS III)	1982	9.27 ± 1.87
Low		340 (17.2)
Medium		1122 (56.6)
High		520 (26.2)
Adolescent health literacy (HLSAC)	1394	31.5 ± 5.13
Low		155 (11.1)
Medium		929 (66.6)
High		310 (22.2)
Parental resilience (BRS-style mean score)	2001	3.43 ± 0.76
Perceived social support (MSPSS)		
MSPSS total score (continuous)	1519	5.83 ± 1.44
Low support (total)		103 (6.8)
Moderate support (total)		158 (10.4)
High support (total)		1258 (82.8)
Family support subscale (mean ± SD)	1537	5.98 ± 1.52
Low family support		109 (5.4)
Moderate family support		139 (6.9)
High family support		1289 (64.3)
Friend support subscale (mean ± SD)	1541	5.57 ± 1.58
Low friend support		134 (6.7)
Moderate friend support		300 (15.0)
High friend support		1107 (55.2)
Significant other support subscale (mean ± SD)	1537	5.88 ± 1.56
Low significant-other support		116 (5.8)
Moderate significant-other support		177 (8.8)
High significant-other support		1244 (62.1)

^1^ Values are reported as n (%) for categorical variables and mean ± standard deviation (SD) for continuous variables. Percentages refer to the total sample unless otherwise indicated; denominators vary owing to item-level missingness. ^2^ The adolescent stage was defined a priori based on age (early adolescence, 11–14 years; late adolescence, 15–19 years). ^3^ Family language reflects the primary language spoken at home, whereas school language indicates the language of instruction. ^4^ Residence was classified as rural or urban based on administrative criteria. Abbreviations: BRS, Brief Resilience Scale; CASMIN, Comparative Analysis of Social Mobility in Industrial Nations; FAS III, Family Affluence Scale, third version; HLSAC, Health Literacy Scale for Adolescents; MSPSS, Multidimensional Scale of Perceived Social Support; SD, standard deviation.

**Table 2:** Distribution of mental well-being indicator scores among adolescents aged 11–19 years.

Well-Being Indicator	Mean ± SD	Observed Range	Valid n
Health-related quality of life (HRQoL, T-values)	50.08 ± 10.08	12–84	1444
Emotional and behavioral difficulties (SDQ total)	8.87 ± 5.62	0–30	1386
Depressive symptoms (PHQ-2)	1.03 ± 1.27	0–6	1438
Anxiety symptoms (SCARED)	5.90 ± 4.65	0–18	1416
Psychosomatic complaints (HBSC-SCL)	3.39 ± 2.31	0–8	1564

Higher scores indicate better health-related quality of life. For all symptom-based measures (SDQ, PHQ-2, SCARED, HBSC-SCL), higher scores indicate greater symptom burden. Sample sizes varied owing to item-level missingness; the valid n for each indicator is shown. Abbreviations: HRQoL, health-related quality of life; HBSC-SCL, Health Behavior in School-aged Children Symptom Checklist; SDQ, Strengths and Difficulties Questionnaire; PHQ-2, Patient Health Questionnaire-2; SCARED, Screen for Child Anxiety-Related Emotional Disorders; SD, standard deviation.

**Table 3:** Associations between parental resilience (BRS mean score) and adolescents’ perceived social support subscales.

Outcome	B (95% CI)	β	*p*	ΔR^2^
MSPSS Family	0.10 (0.00, 0.20)	0.051	0.042	0.002
MSPSS Friends	0.16 (0.06, 0.26)	0.077	0.002	0.006
MSPSS Significant Other	0.18 (0.08, 0.27)	0.086	<0.001	0.007

Linear regression models with each MSPSS subscale as the dependent variable. Unstandardized (B) and standardized (β) coefficients are shown with 95% confidence intervals. ΔR^2^ reflects the incremental variance explained by parental resilience after adjusting for sociodemographic covariates. Missing data were handled using listwise deletion; n = 1639–1649 per model. Abbreviations: MSPSS, Multidimensional Scale of Perceived Social Support.

**Table 4:** Summary of hierarchical regression results: perceived social support and parental resilience as predictors of adolescent mental well-being.

Outcome	R^2^ M1	R^2^ M2	R^2^ M3	B BRS (95% CI)	β	*p*
HRQoL	0.071	0.151	0.174	2.05 (1.43; 2.67)	0.155	<0.001
SDQ	0.020	0.080	0.102	−1.11 (−1.48; −0.74)	−0.149	<0.001
SCARED	0.087	0.104	0.121	−0.84 (−1.14; −0.53)	−0.135	<0.001
PHQ-2	0.086	0.132	0.145	−0.20 (−0.28; −0.12)	−0.115	<0.001
HBSC-SCL	0.070	0.104	0.134	–0.54 (−0.69; 0.39)	–0.176	<0.001

Unstandardized (B) and standardized (β) coefficients for parental resilience (Model 3) with 95% confidence intervals. Detailed coefficients for all predictors are reported in Supplementary Tables S2–S6. Abbreviations are shown in Table 2.

## Data Availability

The data presented in this study are available from the corresponding author upon reasonable request.

## Supplementary Materials

The following supporting information can be downloaded at: https://www.mdpi.com/article/10.3390/children13050615/s1, Table S1: Spearman correlation matrix of parental resilience, perceived social support, and adolescent mental well-being indicators; Table S2: Health-related quality of life (HRQoL; KIDSCREEN-10 T-values) from hierarchical multivariable linear regression; Table S3: Emotional and behavioral difficulties (SDQ total difficulties score) from hierarchical multivariable linear regression; Table S4: Depressive symptoms (PHQ-2 score) from hierarchical multivariable linear regression; Table S5: Anxiety symptoms (SCARED score) from hierarchical multivariable linear regression; Table S6: Psychosomatic complaints (HBSC-SCL number of weekly complaints) from hierarchical multivariable linear regression; Table S7: Mediation analyses: perceived family support (MSPSS family subscale) as mediator of the association between parental resilience (BRS) and adolescent mental well-being outcomes (PROCESS Model 4); Table S8: Moderation analyses: adolescent sex and developmental stage as moderators of the association between parental resilience and mental well-being outcomes (PROCESS Model 1).

## Abbreviations

The following abbreviations are used in this manuscript:

AI	Artificial intelligence
BRS	Brief Resilience Scale
CASMIN	Comparative Analysis of Social Mobility in Industrial Nations
CI	Confidence interval
COP-S	Corona and Psyche—South Tyrol
FAS III	Family Affluence Scale III
HBSC-SCL	Health Behavior in School-aged Children Symptom Checklist
HLSAC	Health Literacy for School-Aged Children
HRQoL	health-related quality of life
MSPSS	Multidimensional Scale of Perceived Social Support
PHQ-2	Patient Health Questionnaire-2
SCARED-GAD	Screen for Child Anxiety-Related Emotional Disorders
SD	Standard deviation
SDQ	Strengths and Difficulties Questionnaire
VIFs	Variance inflation factors
WHO	World Health Organization
