# Supplementary material for: Parental Resilience and Adolescent Mental Well-Being: A Population-Based Study
Source: Children (Basel). 2026 Apr 29;13(5):615. doi: 10.3390/children13050615 (PMC13205128; doi:10.3390/children13050615)
Supplement: Supplementary file 1 [file children-13-00615-s001.zip › children-4266977-supplementary.pdf]

Supplementary Material

Table S1. Spearman rank-order correlation matrix of parental resilience, perceived social support, and adolescent mental well-being indicators.

Variable	1	2	3	4	5	6	7	8	9	10
1. MSPSS total	1									
2. MSPSS family	0.826**	1								
3. MSPSS friends	0.895**	0.584**	1							
4. MSPSS significant others	0.877**	0.746**	0.684**	1						
5. Parental resilience (BRS)	0.119**	0.094**	0.097**	0.118**	1					
6. HRQoL	0.442**	0.412**	0.381**	0.329**	0.151**	1				
7. SDQ total	-0.367**	-0.337**	-0.317**	-0.249**	-0.154**	-0.602**	1			
8. PHQ-2	-0.306**	-0.308**	-0.242**	-0.223**	-0.106**	-0.630**	0.564**	1		
9. SCARED	-0.251**	-0.233**	-0.215**	-0.152**	-0.142**	-0.540**	0.626**	0.544**	1	
10. Psychosomatic complaints (HBSC-SCL)	-0.290**	-0.293**	-0.235**	-0.192**	-0.166**	-0.592**	0.582**	0.575**	0.587**	1

Spearman rank-order correlation coefficients (rs) based on pairwise complete observations. Variable 5 (parental resilience) reflects the Brief Resilience Scale (BRS) mean score administered to parents. All other variables are adolescent self-report. Higher scores indicate higher perceived social support, greater parental resilience, better health-related quality of life, or greater symptom burden depending on the scale. * $p < 0.05$, ** $p < 0.01$ (two-tailed). Abbreviations: BRS, Brief Resilience Scale; HRQoL, health-related quality of life; MSPSS, Multidimensional Scale of Perceived Social Support; PHQ-2, Patient Health Questionnaire-2; SCARED, Screen for Child Anxiety-Related Emotional Disorders; SDQ, Strengths and Difficulties Questionnaire.

Table S2. Health-related quality of life (HRQoL; KIDSCREEN-10 T-values) from hierarchical multivariable linear regression.

Predictor	B [95% CI]	β	*p*-value
Model 1 (sociodemographic covariates only)			
(Constant)	64.82 [60.73; 68.90]	—	< 0.001
Age (years)	-0.99 [-1.21; -0.78]	-0.227	< 0.001
Female sex (ref. male)	-2.11 [-3.09; -1.13]	-0.104	< 0.001
Parental education (CASMIN)	0.13 [-0.16, 0.41]	0.023	0.392
Family affluence (FAS III)	-0.14 [-0.86; 0.58]	-0.010	0.699
Migration background (yes)	-1.29 [-3.07; 0.50]	-0.035	0.157
Single-parent household	-2.16 [-3.77; -0.55]	-0.068	0.009
Urban residence	0.16 [-0.96; 1.28]	0.007	0.781
Perceived social support (MSPSS total)	—	—	—
Parental resilience (BRS mean score)	—	—	—
Model 2 (+ perceived social support, MSPSS total)			
(Constant)	52.83 [48.46; 57.21]	—	< 0.001

Predictor	B [95% CI]	β	*p*-value
Age (years)	-0.95 [-1.15; -0.75]	-0.217	< 0.001
Female sex (ref. male)	-2.30 [-3.24; -1.36]	-0.114	< 0.001
Parental education (CASMIN)	0.13 [-0.15; 0.40]	0.023	0.372
Family affluence (FAS III)	-0.41 [-1.10; 0.28]	-0.030	0.243
Migration background (yes)	-0.88 [-2.59; 0.83]	-0.024	0.314
Single-parent household	-1.62 [-3.17; -0.08]	-0.051	0.040
Urban residence	0.19 [-0.88; 1.26]	0.008	0.734
Perceived social support (MSPSS total)	2.05 [1.71; 2.38]	0.285	< 0.001
Parental resilience (BRS mean score)	—	—	—
Model 3 (+ parental resilience, BRS mean score)			
(Constant)	47.51 [42.90, 52.12]	—	< 0.001
Age (years)	-0.96 [-1.17, -0.76]	-0.220	< 0.001
Female sex (ref. male)	-2.20 [-3.13, -1.27]	-0.109	< 0.001
Parental education (CASMIN)	0.02 [-0.25, 0.30]	0.004	0.869
Family affluence (FAS III)	-0.52 [-1.20, 0.16]	-0.038	0.131
Migration background (yes)	-1.13 [-2.82, 0.56]	-0.031	0.188
Single-parent household	-1.93 [-3.45, -0.40]	-0.061	0.013
Urban residence	0.01 [-1.05, 1.06]	0.000	0.991
Perceived social support (MSPSS total)	1.99 [1.66, 2.32]	0.277	< 0.001
Parental resilience (BRS mean score)	2.05 [1.43, 2.67]	0.155	< 0.001

Unstandardized regression coefficients (B) with 95% confidence intervals and standardized coefficients (β) are shown. Model 1 includes sociodemographic covariates only (age, sex, parental education, family affluence, migration background, household structure, urbanicity). Model 2 additionally includes perceived social support (MSPSS total score). Model 3 further includes parental resilience (BRS mean score). Missing data were handled using listwise deletion. Model fit: $R^2 = 0.071$, $\Delta R^2 = 0.071$, $F(7, 1514) = 16.49$, $p < 0.001$, $n = 1{,}522$ (Model 1); $R^2 = 0.151$, $\Delta R^2 = 0.080$, $F(1, 1513) = 142.70$, $p < 0.001$, $n = 1{,}522$ (Model 2); $R^2 = 0.174$, $\Delta R^2 = 0.023$, $F(1, 1512) = 42.30$, $p < 0.001$, $n = 1{,}522$ (Model 3). Collinearity was low (all VIF < 2.5). Abbreviations: BRS, Brief Resilience Scale; CI, confidence interval; FAS III, Family Affluence Scale III; MSPSS, Multidimensional Scale of Perceived Social Support.

Table S3. Emotional and behavioral difficulties (SDQ total difficulties score) from hierarchical multivariable linear regression.

Predictor	B [95% CI]	β	*p*-value
Model 1 (sociodemographic covariates only)			
(Constant)	6.60 [4.20; 9.00]	—	< 0.001
Age (years)	0.16 [0.03; 0.28]	0.064	0.014
Female sex (ref. male)	1.11 [0.53; 1.69]	0.098	< 0.001
Parental education (CASMIN)	-0.03 [-0.19; 0.14]	-0.008	0.768
Family affluence (FAS III)	-0.21 [-0.63; 0.21]	-0.027	0.334

Predictor	B [95% CI]	β	*p*-value
Migration background (yes)	0.76 [-0.29; 1.81]	0.037	0.156
Single-parent household	0.98 [0.03; 1.92]	0.055	0.042
Urban residence	0.16 [-0.50; 0.82]	0.012	0.641
Perceived social support (MSPSS total)	—	—	—
Parental resilience (BRS mean score)	—	—	—
Model 2 (+ perceived social support, MSPSS total)			
(Constant)	12.40 [9.80; 15.01]	—	< 0.001
Age (years)	0.13 [0.01; 0.25]	0.053	0.035
Female sex (ref. male)	1.18 [0.62; 1.74]	0.104	< 0.001
Parental education (CASMIN)	-0.02 [-0.18; 0.15]	-0.006	0.832
Family affluence (FAS III)	-0.06 [-0.47; 0.35]	-0.008	0.763
Migration background (yes)	0.54 [-0.48; 1.55]	0.026	0.303
Single-parent household	0.72 [-0.19; 1.64]	0.041	0.121
Urban residence	0.08 [-0.56; 0.71]	0.006	0.819
Perceived social support (MSPSS total)	-0.99 [-1.19; -0.79]	-0.246	< 0.001
Parental resilience (BRS mean score)	—	—	—
Model 3 (+ parental resilience, BRS mean score)			
(Constant)	15.30 [12.54; 18.05]	—	< 0.001
Age (years)	0.14 [0.02; 0.26]	0.056	0.026
Female sex (ref. male)	1.11 [0.56; 1.67]	0.098	< 0.001
Parental education (CASMIN)	0.04 [-0.12; 0.21]	0.014	0.608
Family affluence (FAS III)	-0.02 [-0.43; 0.38]	-0.003	0.920
Migration background (yes)	0.67 [-0.33; 1.68]	0.033	0.190
Single-parent household	0.90 [-0.00; 1.81]	0.051	0.051
Urban residence	0.17 [-0.46; 0.81]	0.014	0.589
Perceived social support (MSPSS total)	-0.96 [-1.16; -0.76]	-0.239	< 0.001
Parental resilience (BRS mean score)	-1.11 [-1.48; -0.74]	-0.149	< 0.001

Unstandardized regression coefficients (B) with 95% confidence intervals and standardized coefficients (β) are shown. Model 1 includes sociodemographic covariates only (age, sex, parental education, family affluence, migration background, household structure, urbanicity). Model 2 additionally includes perceived social support (MSPSS total score). Model 3 further includes parental resilience (BRS mean score). Missing data were handled using listwise deletion. Model fit: $R^2 = 0.020$, $\Delta R^2 = 0.020$, $F(7, 1454) = 4.35$, $p < 0.001$, $n = 1{,}462$ (Model 1); $R^2 = 0.080$, $\Delta R^2 = 0.060$, $F(1, 1453) = 94.24$, $p < 0.001$, $n = 1{,}462$ (Model 2); $R^2 = 0.102$, $\Delta R^2 = 0.021$, $F(1, 1452) = 34.56$, $p < 0.001$, $n = 1{,}462$ (Model 3). Collinearity was low (all VIF < 2.5). Abbreviations: BRS, Brief Resilience Scale; CI, confidence interval; FAS III, Family Affluence Scale III; MSPSS, Multidimensional Scale of Perceived Social Support.

Table S4. Depressive symptoms (PHQ-2 score) from hierarchical multivariable linear regression.

**Predictor**	**B [95% CI]**	**β**	***p*-value**
Model 1 (sociodemographic covariates only)			
(Constant)	-1.23 [-1.75; -0.71]	—	< 0.001
Age (years)	0.14 [0.11; 0.16]	0.242	< 0.001
Female sex (ref. male)	0.37 [0.25; 0.50]	0.143	< 0.001
Parental education (CASMIN)	0.01 [-0.03; 0.05]	0.016	0.543
Family affluence (FAS III)	-0.00 [-0.09; 0.09]	-0.001	0.972
Migration background (yes)	0.22 [-0.01; 0.45]	0.046	0.064
Single-parent household	0.20 [-0.01; 0.41]	0.049	0.059
Urban residence	0.01 [-0.14; 0.15]	0.002	0.930
Perceived social support (MSPSS total)	—	—	—
Parental resilience (BRS mean score)	—	—	—
Model 2 (+ perceived social support, MSPSS total)			
(Constant)	-0.07 [-0.64; 0.50]	—	0.802
Age (years)	0.13 [0.10; 0.16]	0.233	< 0.001
Female sex (ref. male)	0.39 [0.27; 0.51]	0.150	< 0.001
Parental education (CASMIN)	0.01 [-0.02; 0.05]	0.017	0.514
Family affluence (FAS III)	0.03 [-0.06; 0.12]	0.015	0.566
Migration background (yes)	0.17 [-0.06; 0.39]	0.036	0.141
Single-parent household	0.14 [-0.06; 0.34]	0.034	0.173
Urban residence	-0.00 [-0.14; 0.14]	-0.001	0.964
Perceived social support (MSPSS total)	-0.20 [-0.24; -0.15]	-0.216	< 0.001
Parental resilience (BRS mean score)	—	—	—
Model 3 (+ parental resilience, BRS mean score)			
(Constant)	0.44 [-0.17; 1.04]	—	0.156
Age (years)	0.13 [0.11; 0.16]	0.235	< 0.001
Female sex (ref. male)	0.38 [0.26; 0.50]	0.147	< 0.001
Parental education (CASMIN)	0.02 [-0.01; 0.06]	0.031	0.236
Family affluence (FAS III)	0.04 [-0.05; 0.12]	0.020	0.425
Migration background (yes)	0.19 [-0.03; 0.42]	0.041	0.087
Single-parent household	0.17 [-0.03; 0.37]	0.042	0.093
Urban residence	0.01 [-0.13; 0.15]	0.005	0.850
Perceived social support (MSPSS total)	-0.19 [-0.23; -0.15]	-0.209	< 0.001
Parental resilience (BRS mean score)	-0.20 [-0.28; -0.12]	-0.115	< 0.001

Unstandardized regression coefficients (B) with 95% confidence intervals and standardized coefficients (β) are shown. Model 1 includes sociodemographic covariates only (age, sex, parental education, family affluence, migration background, household structure, urbanicity). Model 2 additionally includes perceived social support (MSPSS total score). Model 3 further includes parental resilience (BRS mean score). Missing data were handled using listwise deletion. Model fit: $R^2 = 0.086$, $\Delta R^2 = 0.086$, F(7,

1503) = 20.31, $p < 0.001$, $n = 1{,}511$ (Model 1); $R^2 = 0.132$, $\Delta R^2 = 0.046$, $F(1, 1502) = 79.52$, $p < 0.001$, $n = 1{,}511$ (Model 2); $R^2 = 0.145$, $\Delta R^2 = 0.013$, $F(1, 1501) = 22.40$, $p < 0.001$, $n = 1{,}511$ (Model 3). Collinearity was low (all VIF < 2.5). Abbreviations: BRS, Brief Resilience Scale; CI, confidence interval; FAS III, Family Affluence Scale III; MSPSS, Multidimensional Scale of Perceived Social Support.

Table S5. Anxiety symptoms (SCARED score) from hierarchical multivariable linear regression.

Predictor	B [95% CI]	β	*p*-value
Model 1 (sociodemographic covariates only)			
(Constant)	-0.50 [-2.42; 1.42]	—	0.611
Age (years)	0.30 [0.20; 0.40]	0.148	< 0.001
Female sex (ref. male)	2.21 [1.74; 2.67]	0.233	< 0.001
Parental education (CASMIN)	0.08 [-0.06; 0.21]	0.031	0.248
Family affluence (FAS III)	0.02 [-0.32; 0.35]	0.003	0.920
Migration background (yes)	0.30 [-0.54; 1.13]	0.018	0.483
Single-parent household	1.20 [0.44; 1.95]	0.081	0.002
Urban residence	0.27 [-0.25; 0.79]	0.026	0.311
Perceived social support (MSPSS total)	—	—	—
Parental resilience (BRS mean score)	—	—	—
Model 2 (+ perceived social support, MSPSS total)			
(Constant)	2.05 [-0.08; 4.18]	—	0.059
Age (years)	0.30 [0.20; 0.40]	0.144	< 0.001
Female sex (ref. male)	2.24 [1.78; 2.70]	0.237	< 0.001
Parental education (CASMIN)	0.08 [-0.06; 0.21]	0.031	0.251
Family affluence (FAS III)	0.07 [-0.26; 0.41]	0.012	0.661
Migration background (yes)	0.17 [-0.65; 1.00]	0.010	0.678
Single-parent household	1.07 [0.32; 1.83]	0.073	0.005
Urban residence	0.24 [-0.27; 0.76]	0.023	0.356
Perceived social support (MSPSS total)	-0.43 [-0.60; -0.27]	-0.129	< 0.001
Parental resilience (BRS mean score)	—	—	—
Model 3 (+ parental resilience, BRS mean score)			
(Constant)	4.22 [1.97; 6.47]	—	< 0.001
Age (years)	0.30 [0.20; 0.40]	0.147	< 0.001
Female sex (ref. male)	2.20 [1.74; 2.65]	0.232	< 0.001
Parental education (CASMIN)	0.12 [-0.01; 0.25]	0.047	0.078
Family affluence (FAS III)	0.12 [-0.21; 0.45]	0.018	0.488
Migration background (yes)	0.28 [-0.54; 1.10]	0.017	0.498
Single-parent household	1.21 [0.47; 1.96]	0.082	0.001

Predictor	B [95% CI]	β	*p*-value
Urban residence	0.32 [-0.20; 0.83]	0.030	0.225
Perceived social support (MSPSS total)	-0.41 [-0.57; -0.25]	-0.122	< 0.001
Parental resilience (BRS mean score)	-0.84 [-1.14; -0.53]	-0.135	< 0.001

Unstandardized regression coefficients (B) with 95% confidence intervals and standardized coefficients (β) are shown. Model 1 includes sociodemographic covariates only (age, sex, parental education, family affluence, migration background, household structure, urbanicity). Model 2 additionally includes perceived social support (MSPSS total score). Model 3 further includes parental resilience (BRS mean score). Missing data were handled using listwise deletion. Model fit: $R^2 = 0.087$, $\Delta R^2 = 0.087$, $F(7, 1481) = 20.24$, $p < 0.001$, $n = 1{,}489$ (Model 1); $R^2 = 0.104$, $\Delta R^2 = 0.016$, $F(1, 1480) = 27.11$, $p < 0.001$, $n = 1{,}489$ (Model 2); $R^2 = 0.121$, $\Delta R^2 = 0.018$, $F(1, 1479) = 29.48$, $p < 0.001$, $n = 1{,}489$ (Model 3). Collinearity was low (all VIF < 2.5). Abbreviations: BRS, Brief Resilience Scale; CI, confidence interval; FAS III, Family Affluence Scale III; MSPSS, Multidimensional Scale of Perceived Social Support.

Table S6. Psychosomatic complaints (HBSC-SCL number of weekly complaints) from hierarchical multivariable linear regression.

Predictor	B [95% CI[	β	*p*-value
Model 1 (sociodemographic covariates only)			
(Constant)	−0.84 [−1.85; 0.17]	—	0.103
Age (years)	0.15 [0.10; 0.20]	0.152	< 0.001
Female sex (ref. male)	0.89 [0.66; 1.12]	0.192	< 0.001
Parental education (CASMIN)	0.08 [−0.09; 0.24]	0.024	0.376
Family affluence (FAS III)	0.05 [−0.02; 0.11]	0.036	0.186
Migration background (yes)	0.23 [−0.19; 0.64]	0.027	0.289
Single-parent household	0.49 [0.11; 0.87]	0.067	0.011
Urban residence	0.09 [−0.17; 0.35]	0.018	0.488
Perceived social support (MSPSS total)	—	—	—
Parental resilience (BRS mean score)	—	—	—
Model 2 (+ perceived social support, MSPSS total)			
(Constant)	1.05 [−0.05; 2.15]	—	0.062
Age (years)	0.14 [0.10; 0.19]	0.144	< 0.001
Female sex (ref. male)	0.91 [0.69; 1.13]	0.197	< 0.001
Parental education (CASMIN)	0.11 [−0.05; 0.28]	0.036	0.174
Family affluence (FAS III)	0.05 [−0.02; 0.11]	0.038	0.159
Migration background (yes)	0.15 [−0.26; 0.56]	0.018	0.472
Single-parent household	0.43 [0.06; 0.80]	0.059	0.024
Urban residence	0.07 [−0.18; 0.32]	0.014	0.589
Perceived social support (MSPSS total)	−0.32 [−0.40; −0.24]	−0.187	< 0.001
Parental resilience (BRS mean score)	—	—	—
Model 3 (+ parental resilience, BRS mean score)			

Predictor	B [95% CI[	β	*p*-value
(Constant)	2.45 [1.30; 3.60]	—	< 0.001
Age (years)	0.15 [0.10; 0.20]	0.149	< 0.001
Female sex (ref. male)	0.87 [0.65; 1.09]	0.188	< 0.001
Parental education (CASMIN)	0.15 [−0.02; 0.31]	0.046	0.078
Family affluence (FAS III)	0.07 [0.01; 0.14]	0.059	0.027
Migration background (yes)	0.23 [−0.18; 0.63]	0.027	0.270
Single-parent household	0.51 [0.14; 0.88]	0.070	0.007
Urban residence	0.11 [−0.14; 0.36]	0.021	0.399
Perceived social support (MSPSS total)	−0.30 [−0.39; −0.22]	−0.177	< 0.001
Parental resilience (BRS mean score)	−0.54 [−0.69; −0.39]	−0.176	< 0.001

Unstandardized regression coefficients (B) with 95% confidence intervals and standardized coefficients (β) are shown. Model 1 includes sociodemographic covariates only (age, sex, parental education, family affluence, migration background, household structure, urbanicity). Model 2 additionally includes perceived social support (MSPSS total score). Model 3 further includes parental resilience (BRS mean score). Missing data were handled using listwise deletion. Model fit: $R^2 = 0.070$, $\Delta R^2 = 0.070$, $F(7, 1469) = 15.754$, $p < 0.001$, n = 1,477 (Model 1); $R^2 = 0.104$, $\Delta R^2 = 0.035$, $F(1, 1468) = 56.787$, $p < 0.001$, n = 1,477 (Model 2); $R^2 = 0.134$, $\Delta R^2 = 0.030$, $F(1, 1467) = 50.782$, $p < 0.001$, n = 1,477 (Model 3). Collinearity was low (all VIF < 1.21). Abbreviations: BRS, Brief Resilience Scale; CI, confidence interval; FAS III, Family Affluence Scale III; MSPSS, Multidimensional Scale of Perceived Social Support.

Table S7. Mediation analyses: perceived family support (MSPSS family subscale) as mediator of the association between parental resilience (BRS) and adolescent mental well-being outcomes (PROCESS Model 4).

Outcome (Y)	a-path B (SE) BRS→MSPSS-Fam	*p*	b-path B (SE) MSPSS-Fam→Y	*p*	Direct effect B (SE) BRS→Y	*p*	Indirect effect a×b	95% BC Bootstrap CI	Mediation
HRQoL	0.10 (0.10)	0.322	2.05 (0.32)	< 0.001	2.16 (0.43)	< 0.001	0.087	[-0.111; 0.291]	No
SDQ	0.10 (0.10)	0.322	-1.11 (0.19)	< 0.001	-1.16 (0.27)	< 0.001	-0.043	[-0.146; 0.051]	No
PHQ-2	0.09 (0.10)	0.322	-0.20 (0.04)	< 0.001	-0.21 (0.06)	< 0.001	-0.012	[-0.035; 0.007]	No
SCARED	0.08 (0.10)	0.322	-0.84 (0.15)	< 0.001	-0.86 (0.21)	< 0.001	-0.020	[-0.067; 0.019]	No
HBSC-SCL	0.06 (0.05)	0.215	−0.29 (0.04)	< 0.001	−0.54 (0.08)	< 0.001	−0.018	[−0.052; 0.013]	No

Mediation analyses were conducted using the PROCESS macro for SPSS (version 4.3), Model 4. X = parental resilience (BRS mean score); M = perceived family support (MSPSS family subscale); Y = adolescent mental well-being outcome. All models adjusted for adolescent age, sex, parental education, family affluence (FAS III), migration background, household composition, and urbanicity. The a-path represents the association between parental resilience and perceived family support; the b-path, the association between perceived family support and the outcome adjusted for parental resilience; the direct effect, the association between parental resilience and the outcome after adjusting for the mediator. Indirect effects were estimated using bias-corrected bootstrap confidence intervals (5,000 resamples). Mediation is supported when the 95% BC bootstrap CI excludes zero. *n* = 1,467–1,527 per model due to listwise deletion. Abbreviations: BC, bias-corrected; BRS, Brief Resilience Scale; CI, confidence interval; MSPSS, Multidimensional Scale of Perceived Social Support; SE, standard error.

Table S8. Moderation analyses: adolescent sex and developmental stage as moderators of the association between parental resilience and mental well-being outcomes (PROCESS Model 1).

Outcome (Y)	BRS main effect B (SE)	*p*	Moderator main effect B (SE)	*p*	Inter-action B (SE) BRS × Moderator	*p*	ΔR²	F (inter-action)	*p*
Block A: Moderator = Adolescent sex (female = 1, male = 0)									
HRQoL	2.64 (0.47)	0.000	0.56 (2.31)	0.807	-0.74 (0.65)	0.256	0.0008	1.2895	0.256
SDQ	-1.38 (0.28)	0.000	-0.25 (1.36)	0.854	0.37 (0.38)	0.336	0.0006	0.9264	0.336
SCARED	-1.09 (0.22)	0.000	0.72 (1.09)	0.505	0.41 (0.31)	0.176	0.0011	1.8337	0.176
PHQ-2	-0.23 (0.06)	0.000	0.22 (0.30)	0.459	0.04 (0.08)	0.645	0.0001	0.2124	0.645
HBSC-SCL	−0.63 (0.24)	0.009	0.71 (0.53)	0.182	0.04 (0.15)	0.773	0.0000	0.0831	0.773
Block B: Moderator = Developmental stage (late adolescence = 1, early = 0)									
HRQoL	2.35 (0.46)	0.000	-1.13 (2.48)	0.647	-0.15 (0.65)	0.821	0.0000	0.0510	0.821
SDQ	-1.05 (0.27)	0.000	1.01 (1.47)	0.492	-0.28 (0.38)	0.461	0.0004	0.5448	0.461
SCARED	-0.73 (0.22)	0.001	1.04 (1.17)	0.373	-0.29 (0.31)	0.345	0.0005	0.8918	0.345
PHQ-2	-0.18 (0.06)	0.003	0.31 (0.32)	0.335	-0.07 (0.08)	0.415	0.0004	0.6650	0.415
HBSC-SCL	−0.61 (0.11)	< 0.001	0.05 (0.57)	0.933	0.10 (0.15)	0.497	0.0003	0.4608	0.497

Moderation analyses were conducted using the PROCESS macro for SPSS (version 4.3), Model 1. X = parental resilience (BRS mean score); W = moderator (sex or developmental stage); Y = adolescent mental well-being outcome. Block A covariates: adolescent age, parental education, family affluence, migration background, household composition, urbanicity. Block B covariates: as Block A plus adolescent sex. ΔR^2 reflects the incremental variance explained by the interaction term. None of the interaction terms reached statistical significance (all $p \geq 0.176$), indicating no evidence of moderation by sex or developmental stage. $n \approx$ 1,467–1,527 per model. Abbreviations: BRS, Brief Resilience Scale; ΔR^2, change in explained variance.
